# Investigating the Muscular and Kinematic Responses to Sudden Wrist Perturbations During a Dynamic Tracking Task

**DOI:** 10.1038/s41598-020-61117-9

**Published:** 2020-03-05

**Authors:** Garrick N. Forman, Davis A. Forman, Edwin J. Avila-Mireles, Jacopo Zenzeri, Michael W. R. Holmes

**Affiliations:** 1 Faculty of Applied Health Sciences, Brock University, St. Catharines, ON Canada; 2 Faculty of Science, University of Ontario Institute of Technology, Oshawa, ON Canada; 3 Robotics, Brain and Cognitive Sciences, Istituto Italiano di Tecnologia, Genoa, Italy

**Keywords:** Electromyography - EMG, Neurophysiology, Skeletal muscle

## Abstract

Sudden disturbances (perturbations) to the hand and wrist are commonplace in daily activities and workplaces when interacting with tools and the environment. It is important to understand how perturbations influence forearm musculature and task performance when identifying injury mechanisms. The purpose of this work was to evaluate changes in forearm muscle activity and co-contraction caused by wrist perturbations during a dynamic wrist tracking task. Surface electromyography was recorded from eight muscles of the upper-limb. Participants performed trials consisting of 17 repetitions of ±40° of wrist flexion/extension using a robotic device. During trials, participants received radial or ulnar perturbations that were delivered during flexion or extension, and with known or unknown timing. Co-contraction ratios for all muscle pairs showed significantly greater extensor activity across all experimental conditions. Of all antagonistic muscle pairs, the flexor carpi radialis (FCR)-extensor carpi radialis (ECR) muscle pair had the greatest change in co-contraction, producing 1602% greater co-contraction during flexion trials than during extensions trials. Expected perturbations produced greater anticipatory (immediately prior to the perturbation) muscle activity than unexpected, resulting in a 30% decrease in wrist displacement. While improving performance, this increase in anticipatory muscle activity may leave muscles susceptible to early-onset fatigue, which could lead to chronic overuse injuries in the workplace.

## Introduction

Musculoskeletal disorders (MSDs) are among the most common types of workplace injuries in Canada and the USA, accounting for over 40% and 29% of all lost-time claims, respectively^1,2^. Additionally, in Europe, upper extremity MSDs, along with low back pain, account for 39% of occupational diseases^2^. In 2016, illnesses and injuries of the upper extremities accounted for 21.8% of all MSD lost time claims in Canada^1^. To minimize musculoskeletal injury risk, an upper limit of 1–5% of maximum voluntary exertion (MVE) has been proposed for individuals performing continuous work throughout the day^3,4^. This upper limit threshold is difficult to maintain when interacting with tools or objects in the environment. In addition, sudden disturbances (perturbations) to the hand and wrist (distal upper extremity) are routine in daily activities and workplaces when interacting with tools or performing complex tasks. Perturbation protocols allow for insight into how the central nervous system (CNS) provides modulation of joint stiffness when encountering instability. This approach can improve our understanding of muscular demands and adaptations which can advance our understanding of musculoskeletal injury mechanisms.

The CNS can employ numerous control strategies when encountering instability in order to maintain control of joint movement. The neuromuscular system is able to selectively increase joint stiffness and stability by increasing muscular co-contraction^5^. Pre-emptively increasing joint stiffness via co-contraction is metabolically wasteful as muscles are activated before joint stiffness is required^6^. However, attempting to deal with a perturbation post-disturbance may have negative consequences, as the neuromuscular delay associated with a reflex response may lead to injury^7^. Adaptation to instability can be achieved through development of an internal model of the forces being encountered^8^. By developing an internal model, rather than utilizing metabolically inefficient co-contraction, the CNS has the ability to adapt and selectively alter joint impedance based on the magnitude and direction of instability^8^. Developing an internal model to learn optimal joint impedance is not always required to manage instability; Crevecoeur *et al*. (2019) explored a model-free strategy, reporting that encountering a single unexpected perturbation led to an immediate increase in co-contraction followed by a gradual decrease during subsequent, perturbation-free trials^9^. When a wrist perturbation is expected but timing is unknown, anticipatory co-contraction is significantly greater than when the timing is known^10^. These findings could be a reflection of the methodology, since participants knew that a perturbation trial would occur, but were unaware of the timing. At the wrist joint, this co-contraction is illustrated by the stabilizing nature of the wrist extensors, which remain active when performing static tasks, even when they are not the primary muscles involved^11–13^. This leads to an optimization problem for the CNS as control of impedance is computationally costly^8^, while co-contraction is metabolically costly^6^. Therefore, to optimize control, the CNS is required to provide adequate impedance relative to the magnitude and direction of instability while utilizing minimal metabolic fuel^8,9^.

Research utilizing unexpected perturbations at the distal upper extremity has largely been focused around static and/or pre-loading tasks^5,10,14,15^. However, these conditions are often not reflective of many real-world tasks and workplaces. Muscular responses to static conditions and perturbations may not directly transfer to dynamic or complex tasks. Assessing perturbations while performing a dynamic task will provide insights into motor and muscular strategies utilized to maintain performance while minimizing the impact of the perturbing force. A number of studies have explored joint stability and impedance during both static/isometric loading and movement^8,16–20^. It is suggested that the ability to alter joint impedance is greater during movement than under static conditions^16^. Elevated levels of stiffness have been observed during both lateral and longitudinal movement compared to static conditions, specifically, perpendicular to the direction of movement^16^. However, perturbation research at the wrist has only been investigated using forces in the flexion and extension directions with little investigation into the effects of radial and ulnar perturbations. The wrist has a greater radial deviation moment than ulnar deviation^21^, which, in addition to muscle geometry, may lead to varying control strategies based on the direction of instability. Additionally, elevated levels of co-contraction have been observed as movement accuracy demands increase^22,23^. Based on these differences, it could be expected that perturbations during dynamic movement would elicit less displacement about the joint as the muscles are already co-contracting to produce movement and are better able to adjust impedance and increase co-contraction to provide accurate movements. Therefore, the purpose of this study was to determine how radial and ulnar perturbations at the wrist joint effect forearm muscle activity, co-contraction, and performance during a dynamic tracking task. We hypothesized that: (1) For perturbation timing and knowledge variables - greater co-contraction would be observed in the anticipatory time period prior to an unexpected perturbation when compared to an expected perturbation. It is hypothesized that a potential control strategy would be to stiffen the wrist joint (via increased co-contraction) continuously throughout the unexpected trial. When the perturbation is expected, participants may focus on controlling the handle and compensate by dealing with the perturbation in the post phase. (2) For the wrist extensors - greater co-contraction would be produced by the wrist extensors when compared to the wrist flexors in order to stabilize the wrist during all perturbation conditions. (3) For perturbation direction variables - radial perturbations will result in greater muscle activity from muscles which generate ulnar deviation.

## Methods

### Participants

Twelve right-handed, male participants (Age: 24 ± 2 years; Height: 177 ± 8 cm; Weight: 83 ± 15 kg) were recruited for this study. Participants were excluded from participating if they had any upper limb or neurological injuries, or reduced mobility due to prior injury. This study was approved by the Brock University Research Ethics Board (REB# 16–262) and performed in accordance with the committee. Once all questions were addressed, participants read and signed an informed consent.

### Experimental setup

Participants were familiarized with the robotic device used for the experiment (WristBot, Genoa, Liguria, Italy)^24^. The WristBot moves with 3 degrees of freedom and with the following ranges of motion: Flexion/Extension = ±62°; Radial/Ulnar Deviation = +45°/−40°; Pronation/Supination = ± 60°^25^. By manipulating the handle of the WristBot, participants were asked to track a cursor on a computer monitor for two trials, totaling 30 repetitions, to become familiar with the device. Both trials involved a flexion/extension motion with participants receiving either a radial or ulnar perturbation during every repetition. This allowed participants to become familiar with the magnitude and timing of the perturbations they would receive during the experimental trials. The purpose of this was to normalize the participant’s reaction to the perturbations during data collection and minimize over-reactions to receiving a perturbation. It also allowed for participants to acclimate to the robotic device and the on-screen tracking as the WristBot is a novel piece of equipment (further described below).

Once participants completed the familiarization, they were prepared for muscle activity collection. Following standard skin preparation, surface electromyography (EMG) electrodes (MediTrace 130, Kendall, Mansfield, MA, USA) were placed over the muscle bellies of eight muscles on the participant’s dominant upper extremity. Muscles included: flexor carpi radialis (FCR), flexor carpi ulnaris (FCU), flexor digitorum superficialis (FDS), extensor carpi radialis (ECR), extensor carpi ulnaris (ECU), extensor digitorum communis (EDC), brachioradialis (BR), and biceps brachii (BB). Electrode placements followed guidelines outlined by^26^, electrodes were placed in line with muscle fiber orientation and had an inter-electrode distance of 2.5 cm. EMG signals were band-pass filtered (10–1000 Hz) and differentially amplified (CMRR > 115 dB at 60 Hz; input impedance, ~10 GV; AMT-8, Bortec Biomedical Ltd, Calgary, AB Canada). Following electrode placement, signal quality and gain was reviewed and adjusted using a digital oscilloscope (LabView 2016, National Instruments, Austin, TX, USA).

Participants performed a maximum grip trial used to assess muscular fatigue following the experimental protocol (MIE Medical Research Ltd, Leeds, Yorkshire, UK). EMG and grip force data were sampled at 2000 Hz (USB-6229 BNC, National Instruments, Austin, TX, USA). Following the maximum grip trial, participants performed eight, muscle specific maximum voluntary contractions (MVCs) to obtain maximum voluntary excitation (MVE). The researcher manually resisted the participant during MVC trials in accordance with previous literature (see Table 1 of Forman *et al*., 2019 for details). MVE data was used to later normalize the muscle activity from experimental trials.

**Table 1:** Experimental trial conditions.

Timing	Known Timing				Unknown Timing			
Wrist motion	Flexion		Extension		Flexion		Extension	
Perturbation Direction	Radial	Ulnar	Radial	Ulnar	Radial	Ulnar	Radial	Ulnar

Participants performed 8 experimental trials. Trials involved all possible combinations of the three experimental variables.

**Figure 1:**
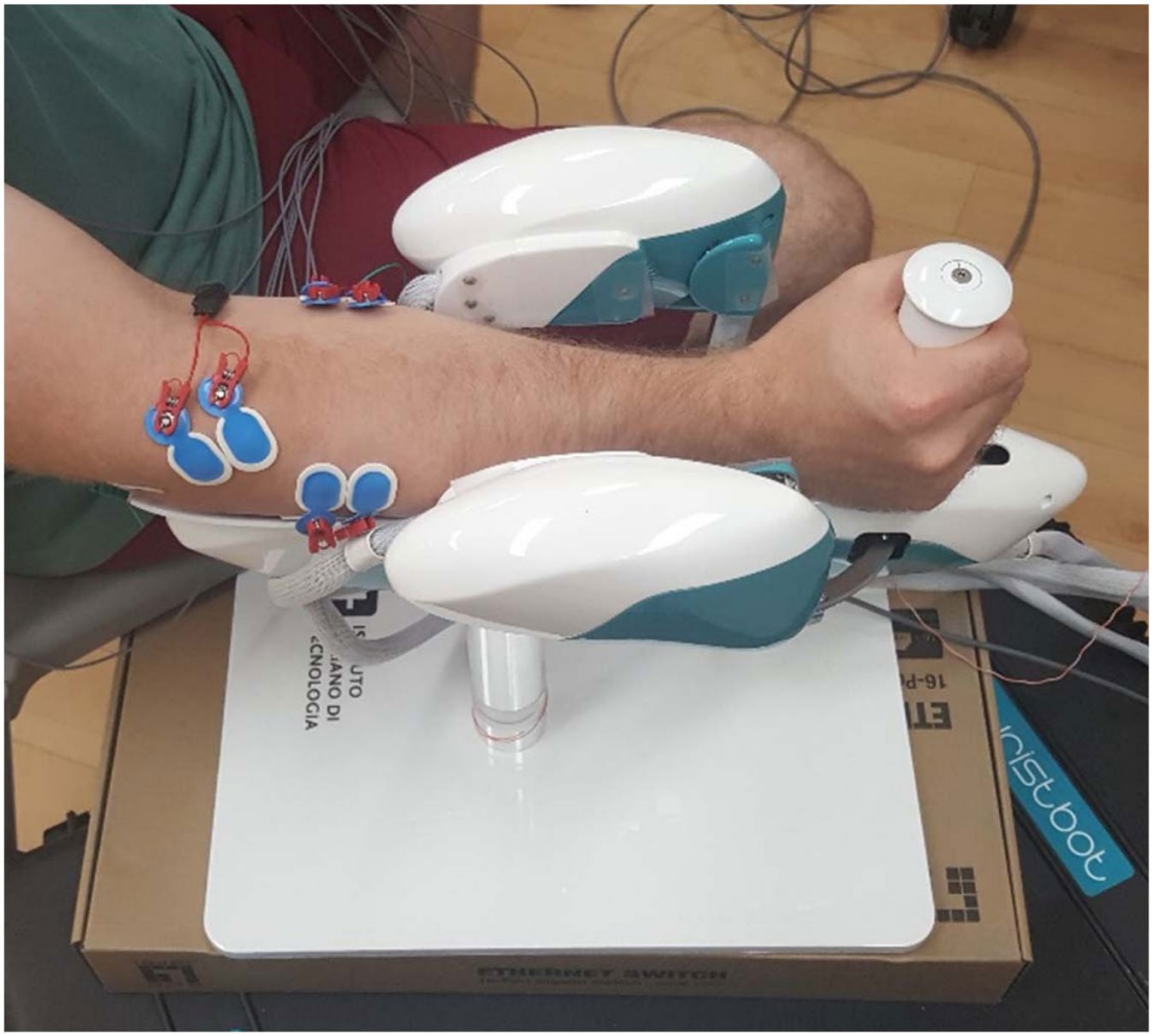
Participant positioned on the Wristbot with EMG electrodes placed over muscles of interest.

Participants were then seated next to the WristBot with their dominant arm placed on the armrest (Fig. 1) and upper arm positioned in the scapular plane. Participants had their anatomically neutral wrist position marked on the robot to normalize the starting position for each trial. Participant’s shoulder and elbow positions were not normalized; however, elbow flexion, shoulder abduction, and shoulder flexion angles were measured and recorded using a goniometer (elbow flexion: 137 ± 7°; shoulder abduction: 35 ± 7°; shoulder flexion: 25 ± 5°). EMG and the WristBot kinematics were time-synchronized with a digital trigger (USB-6229 BNC, National Instruments, Austin, TX, USA). Information about the timing of the perturbation was sent from the robot to the EMG data acquisition through the trigger at 1 kHz. During experimental trials, the WristBot collected the participant’s hand positional data in three axes (flexion/extension, radial/ulnar deviation, and pronation/supination). Kinematic data was sampled at 100 Hz. This provided insight into how accurately participants followed a target as well as any affects the perturbations may have had on the performance.

### Experimental trials

**Figure 2:**
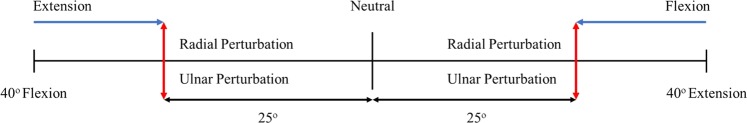
Schematic of experimental trials. Participants moved through ±40° of flexion and extension for each repetition. Perturbations were delivered 25° from neutral, as participants began to flex or extend the wrist (indicated by blue arrows). Perturbations either caused radial or ulnar deviation (indicated by red arrows).

Participants were required to follow an onscreen cursor for 17 repetitions, moving through a range of motion of 40° of flexion and 40° of extension with a frequency of 0.25 Hz. The WristBot compensated for gravity and handle mass, but no resistance or assistance occurred during the flexion/extension movement. Participants performed eight experimental trials in a fully randomized order. Experimental trials included all possible combinations of the experimental variables. Trial variables included: (1) Timing - known and unknown perturbation timing, (2) Phase - perturbations delivered while moving into wrist flexion or extension, and (3) Direction - perturbations delivered in either the radial or ulnar direction (outlined in Table 1). During each trial, three perturbations were delivered at wrist angles ±25° from neutral; perturbations varied based on the specified trial parameters (Fig. 2). The WristBot delivered a 1.54 Nm torque over 200 ms causing radial or ulnar deviation of the wrist. Perturbation magnitude was consistent with previous perturbation work by Goodin & Aminoff^11^ and Holmes *et al*.^10^ who utilized perturbing forces of 18.2 N and 18.4 N, respectively. Torque profile from the WristBot consisted of an increase from baseline to 1.54 Nm over 10 ms, sustained for 200 ms, and a return to baseline over 10 ms. Perturbation duration was chosen to elicit angular displacement of the wrist. For known perturbation timing, perturbations were delivered on the 5^th^, 10^th^, and 15^th^ repetitions. During these trials, a researcher counted the number of repetitions for the participant to allow participants to focus solely on tracking the onscreen cursor. For trials with unknown perturbation timing, participants were told what phase of the repetition (flexion or extension) as well as what direction (radial or ulnar) the perturbations would occur. However, participants were unaware of what repetitions the three perturbations would occur. This was to provide participants with equal knowledge and eliminate any variance in pattern recognition between participants. All participants were instructed to trace the onscreen cursor as closely as possible, to resist the perturbation to the best of their ability, and to quickly return to the cursor pathway. Participants received two minutes of rest between each trial to prevent any influence of muscular fatigue.

Following the final experimental trial, participants received two minutes of rest and then performed a maximum grip trial. This was compared to the pre-test maximal grip trial to determine if participants had fatigued during the experimental trials.

### Data analysis

**Figure 3:**
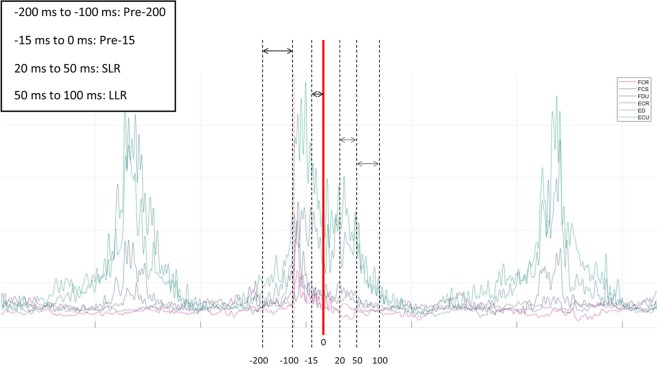
Sample EMG data of six forearm muscles. Outline of time periods used for EMG analysis. Pre-200: −200 ms to −100 ms prior to perturbation; Pre-15: 15 ms prior to perturbation; Short-Latency Reflex (SLR): 20–50 ms following perturbation; Long-Latency Reflex (LLR): 50–100 ms following perturbation. Note: Time periods outlined are visual representations and do not accurately display time points used.

Muscle activity (sample data shown in Fig. 3) from all eight muscles were full-wave rectified and root mean squared. Mean muscle activity was examined during four time periods: Pre-200 (200–100 ms pre-perturbation), Pre-15 (15–0 ms pre-perturbation), short-latency reflex (SLR; 20–50 ms post-perturbation), and long-latency reflex (LLR; 50–100 ms post-perturbation)^10,27–29^.

Co-contraction ratios (CCR) were calculated for antagonistic muscle pairs (FCR-ECR, FDS-EDC, and FCU-ECU). CCRs were computed using an antagonist/agonist ratio depending on which phase of movement the participant was perturbed (e.g. Perturbations during flexion: CCR = extensor/flexor; Perturbations during extension: CCR = flexor/extensor).

$${\rm{CCR}}=\frac{{\rm{Antagonist}}}{{\rm{Agonist}}}$$

**Figure 4:**
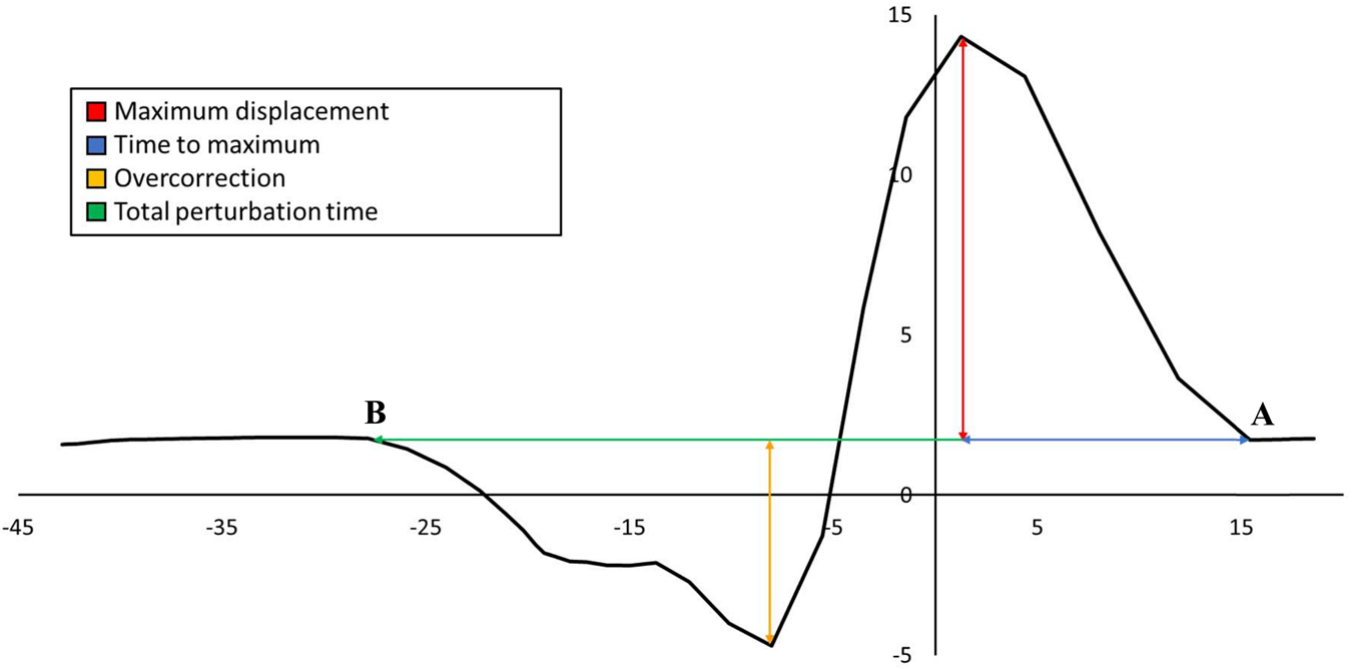
Sample kinematic tracking data of a radial perturbation received during flexion. ‘A’ indicates the point chosen as initial deviation caused by the perturbations and ‘B’ indicates point chosen as return to the target trajectory. Coloured arrows correspond to the legend for kinematic measures.

Using the robot’s kinematic data, participant tracking performance was assessed across all experimental conditions (Fig. 4). Maximum angular displacement (degrees) and time to maximum displacement (milliseconds) were measured from perturbation onset to peak displacement. Overcorrection was measured from maximum negative displacement (opposite direction of perturbation) to return to sustained neutral wrist position. Finally, total perturbation time was measured from perturbation onset to return to a neutral wrist position following radial/ulnar deviation. All kinematic measures were identified using a semi-automated approach by the investigator. A set of objective criteria was used to determine initial wrist deviation and end of wrist deviation. Maximum displacement and overcorrection were not open to interpretation and were simply the maximum and minimum values found between the start and end of wrist deviation. Initial wrist deviation was identified as the data point that the participant deviated from their steady state trajectory. The end of wrist deviation was identified as the data point participants returned to a steady state trajectory.

### Statistical analysis

Mean muscle activity and CCR for each muscle/muscle pair were assessed using a 2 (Knowledge of timing) × 2 (Phase of repetition) × 2 (Direction of perturbation) × 4 (Time period) factorial ANOVA with repeated measures, to evaluate the effects and interactions of the independent variables on mean muscle activity and CCRs. As this work was largely exploratory, this statistical design was chosen to maximize the amount of information obtained regarding the interactions between the independent variables and provided insight into our three primary hypotheses.

A 2 (Knowledge of timing) × 2 (Phase of repetition) × 2 (Direction of perturbation) factorial ANOVA with repeated measures was performed to evaluate the effects and interactions of the independent variables on the maximum angular displacement at the wrist joint, time to reach maximum angular displacement, magnitude of overcorrection when returning to the target path, and total time to return to the target path following a perturbation.

All significant four- and three-way interactions were investigated using simple interactions. Significant two-way interactions were investigated using simple main effects. Pairwise comparisons were performed using a Bonferroni correction.

All results are expressed as Mean ± SD in text while error bars in figures represent standard error. Significance was set to p < 0.05 and determined using IBM SPSS Statistics for Windows, Version 20 (IBM Corp, Armonk, NY, USA).

## Results

Tables 2 and 3 summarize the main effects and interactions, respectively, for each muscle. Effect sizes for muscle activity, calculated using partial eta squared (η_p_^2^), can be found in Tables 4 and 5.

**Table 2:** Significant main effects for muscle activity.

Main Effect P-values				
Muscle	Direction	Knowledge	Phase	Time
FCR	0.324	0.038*	0.001*	0.014*
FDS	0.068	0.089	0.001*	0.012*
FCU	0.121	0.808	0.021*	0.031*
ECR	0.000*	0.151	0.138	0.002*
EDC	0.035*	0.163	0.016*	0.048*
ECU	0.170	0.282	0.011*	0.009*
BR	0.000*	0.045*	0.328	0.017*
BB	0.012*	0.134	0.008*	0.014*

Note: * = statistically significant, p < 0.05.

**Table 3:** Significant interactions for muscle activity.

Interaction P-values											
Muscle	D*K	D*P	K*P	D*K*P	D*T	K*T	D*K*T	P*T	D*P*T	K*P*T	D*K*P*T
FCR	0.451	0.85	0.261	0.407	0.108	0.159	0.275	0.971	0.429	0.209	0.311
FDS	0.595	0.553	0.639	0.942	0.360	0.043*	0.611	0.781	0.310	0.352	0.122
FCU	0.755	0.603	0.386	0.988	0.616	0.031*	0.785	0.528	0.527	0.276	0.956
ECR	0.255	0.350	0.192	0.432	0.002*	0.109	0.238	0.212	0.824	0.019*	0.452
EDC	0.646	0.180	0.133	0.312	0.171	0.052	0.644	0.575	0.066	0.237	0.519
ECU	0.449	0.647	0.884	0.216	0.078	0.007*	0.088	0.237	0.662	0.200	0.723
BR	0.628	0.447	0.167	0.324	0.010*	0.296	0.529	0.137	0.037*	0.044*	0.880
BB	0.913	0.117	0.364	0.042*	0.220	0.236	0.151	0.417	0.493	0.544	0.363

Note: * = statistically significant, p < 0.05.

Note: D = direction; K = knowledge; P = phase; T = time.

**Table 4:** Effect sizes for all EMG main effects.

Effect Sizes (Main Effects)				
Muscle	Direction	Knowledge	Phase	Time
FCR	0.088	0.335	0.620	0.678
FDS	0.271	0.240	0.679	0.685
FCU	0.204	0.006	0.396	0.609
ECR	0.799	0.178	0.188	0.782
EDC	0.343	0.169	0.421	0.567
ECU	0.164	0.104	0.460	0.704
BR	0.721	0.318	0.087	0.662
BB	0.454	0.192	0.489	0.677

**Table 5:** Effect sizes for all EMG interactions.

Effect Sizes (Interactions)											
Muscle	D*K	D*P	K*P	D*K*P	D*T	K*T	D*K*T	P*T	D*P*T	K*P*T	D*K*P*T
FCR	0.053	0.003	0.113	0.063	0.474	0.422	0.366	0.025	0.253	0.381	0.315
FDS	0.026	0.033	0.021	0.001	0.288	0.577	0.175	0.108	0.315	0.292	0.458
FCU	0.009	0.025	0.069	0.000	0.173	0.610	0.107	0.209	0.209	0.335	0.033
ECR	0.116	0.080	0.149	0.057	0.791	0.473	0.360	0.379	0.091	0.653	0.243
EDC	0.020	0.157	0.193	0.093	0.411	0.558	0.162	0.189	0.533	0.361	0.213
ECU	0.053	0.020	0.002	0.135	0.514	0.724	0.500	0.361	0.154	0.388	0.131
BR	0.022	0.054	0.166	0.088	0.696	0.323	0.209	0.443	0.593	0.577	0.069
BB	0.001	0.209	0.075	0.326	0.373	0.362	0.430	0.259	0.224	0.202	0.286

Note: D = direction; K = knowledge; P = phase; T = time.

### Grip force

There was no change in maximum grip force before and after the experimental trials (Pre: 519 ± 106 N; Post: 513 ± 96 N, p = 0.54).

### Muscle activity

**Figure 5:**
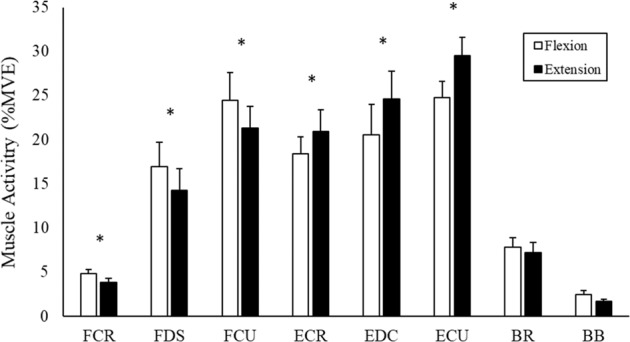
Group averages of muscle activity (%MVE) of the 3 perturbing repetitions, across all time periods, separated into flexion and extension trials. Data includes all time periods collapsed into a single %MVE value. * denotes significant difference between flexion and extension trials.

As anticipated, all six forearm muscles demonstrated greater activity (p < 0.05) throughout the study when they were functioning as the agonist/prime mover versus when they were acting as the antagonist (i.e. the three wrist flexors were more active in wrist flexion trials than extension trials, and vice-versa for the three wrist extensors). Neither BR nor BB showed any differences in muscle activity between movement directions. This was also expected given the low force of movement used in this study and that neither BR nor BB have a direct line of action at the wrist. These results can be seen in Fig. 5.

**Figure 6:**
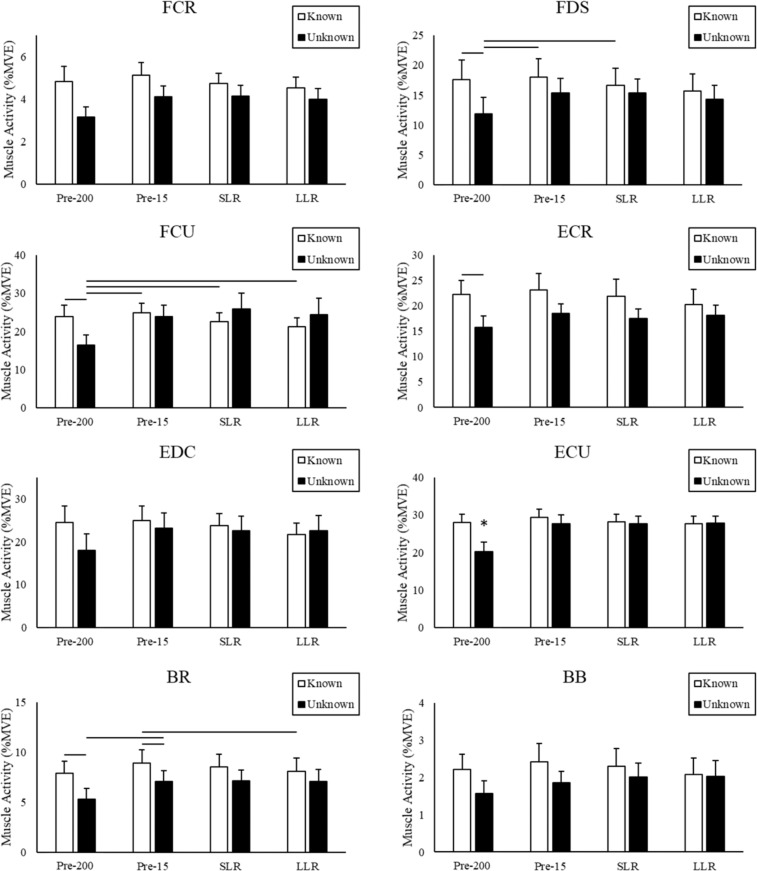
Group averages of muscle activity (%MVE) across all time periods for perturbations with known and unknown timing. Interaction effect was investigated with post-hoc pairwise comparisons. Lines denote significant differences. * denotes significant differences between all other conditions.

Muscle activity responses to known and unknown perturbations proved to be muscle-specific (group averages, across all four time points, are shown in Fig. 6). Although muscle activity tended to be equal or greater during perturbations with known timing for all muscles, only FCR and BR showed a significant main effect (greater EMG during known timing than unknown, p < 0.05). Of the remaining muscles, the largest difference occurred at the Pre-200 time point, with FDS, FCU, ECR, ECU, and BR showing greater EMG during known timing than unknown (p < 0.05). All eight muscles showed a main effect of time (Table 2), with Pre-200 exhibiting less muscle activity than the other three time points (although this only occurred in the unknown trials, Fig. 6).

**Figure 7:**
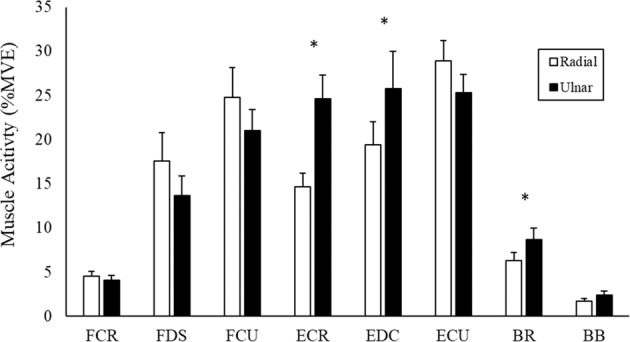
Group averages of muscle activity (%MVE) for the 3 perturbing repetitions, across all time periods, separated by radial and ulnar perturbations. Data includes all time periods collapsed into a single %MVE value. * denotes significant difference between radial and ulnar perturbations.

Perturbation direction (radial perturbations versus ulnar perturbations) significantly influenced muscle activity in the upper-limb, although, like knowledge, this effect was somewhat muscle specific (group averages shown in Fig. 7). While the three wrist flexors (FCR, FDS, and FCU) all trended towards greater muscle activity during radial perturbations (p range of 0.068–0.324), there was no significant difference. Of the three wrist extensors, ECR and EDC exhibited significantly greater muscle activity during ulnar perturbations versus radial (p < 0.05). This finding was shared in the BR and BB, which were also more active in radial perturbations. Perturbation direction had no significant influence on ECU muscle activity, although average EMG did trend towards being greater during ulnar perturbations (p = 0.17).

### Co-contraction ratios

**Figure 8:**
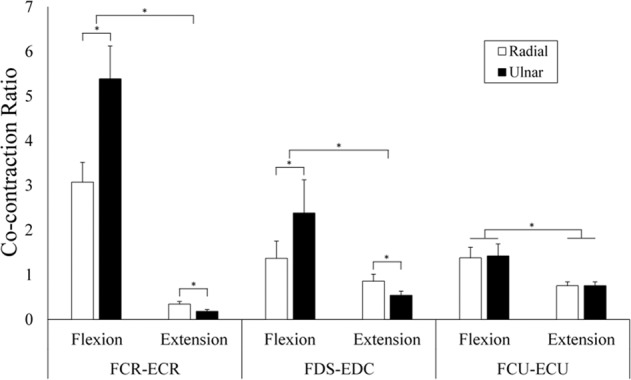
Co-contraction ratios for radial and ulnar perturbations during flexion and extension trials. Flexion ratios were calculated using a extensor/flexor ratio, extension trials were calculated using a flexor/extensor ratio. * denotes significant difference.

Figure 8 depicts antagonist-agonist co-contraction ratios for all anatomical pairs of the forearm muscles. During wrist flexion trials, the ratios were calculated as “Extensor EMG/Flexor EMG,” whereas for wrist extension trials, ratios were calculated as “Flexor EMG/Extensor EMG.” For all three muscle pairings, there was significantly greater co-contraction during wrist flexion conditions versus wrist extension conditions (p < 0.05). This manifested regardless of the perturbation direction (radial or ulnar). Perturbation direction separately influenced co-contraction ratios in the FCR-ECR and FDS-EDC muscle pairings, although this finding was dependent on the direction of wrist movement. For wrist flexion trials, co-contraction was significantly greater during ulnar perturbations than radial perturbations for both FCR-ECR and FDS-EDC (p < 0.05). In wrist extension trials, this relationship reversed, and co-contraction ratios were significantly greater during radial perturbations (p < 0.05). For the FCU-ECU muscle pairing, perturbation direction appeared to have no influence on co-contraction values, regardless of the direction of wrist movement.

### Kinematics

**Figure 9:**
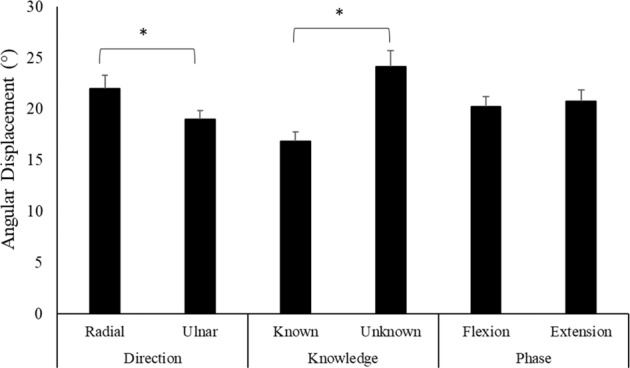
Maximum angular displacement between experimental variables. Statistical comparisons were performed within each variable, no comparisons were investigated between variables. * denotes significant difference.

Figure 9 depicts the angular displacement of the WristBot handle following perturbations in two directions (radial and ulnar), perturbations with known and unknown timing, and perturbations during either flexion or extension wrist movement. Results demonstrated that radial perturbations produced greater maximal angular displacement than ulnar perturbations (Radial: 22.01 ± 4.75°; Ulnar: 19.02 ± 2.91°, p < 0.05). Unsurprisingly, the angular displacement of the handle was significantly greater when participants were perturbed with unknown timing versus when the time of the perturbation was known (Unknown: 24.15 ± 5.40°; Known: 16.87 ± 3.22°, p < 0.05). There was also a significant main effect of perturbation knowledge on time to maximum angular displacement. Perturbations with unknown timing took longer to reach maximum angular displacement than perturbations with known timing (Unknown: 199 ± 5 ms; Known: 181 ± 5 ms, p < 0.05). Lastly, the direction of wrist movement (either wrist flexion or wrist extension) had no influence on angular displacement following a perturbation.

No significant differences in overcorrection were found between trials. For all conditions, participants demonstrated an overcorrection of 5.2 ± 3.4 ° in the direction opposite to the perturbing force.

## Discussion

Sudden disturbances (perturbations) to the hand and wrist are commonplace in activities of daily living and when performing manual labour jobs that require interacting with tools and objects in the environment. Perturbation protocols allow for insight into how the CNS provides modulation of joint stiffness when encountering instability and a better understanding of these processes with common workplace challenges (perturbation timing, knowledge and direction) can aid in future workplace safety standards. Previous work utilizing perturbations at the wrist focused on static and/or pre-loading tasks. However, these conditions are not reflective of real-world tasks and workplaces. Thus, the current study focused on knowledge of perturbation, direction of perturbation and timing of perturbation (known, unknown, posture at delivery) as each variable has unique influences on how the system adapts to the perturbation. Unique insights that can have consequences for better understanding injury mechanisms are provided based on each variable and is discussed below.

### Influence of movement phase

All wrist flexors produced significantly greater activity when perturbed during flexion than extension (Fig. 5). The extensors demonstrated a similar pattern, with all extensors producing greater activity when perturbed during extension. The task dependent nature of the wrist flexors becomes apparent when observing the co-contraction ratios between antagonistic muscle pairs (Fig. 8). All muscle pairings displayed significantly greater co-contraction during flexion trials. FCR-ECR had 1602% greater co-contraction during flexion trials compared to extension trials, with FDS-EDC and FCU-ECU muscle pairs producing 268% and 185% greater co-contraction, respectively. In agreement with our second hypothesis, this indicates that the wrist extensors were active at a higher percentage of maximum than the flexors, regardless of the phase of movement when perturbed. These findings are in line with previous distal upper extremity research investigating forearm muscle actions. Mogk and Keir (2003) found that wrist flexor activity was greatly dependent on posture and grip force requirements, while wrist extensors displayed higher levels of activity that was consistent across all postures. Forman and colleagues (2019) investigated the effects of various grip forces in combination with wrist exertions. The wrist flexors demonstrated low muscle activity during wrist extension while the wrist extensors remained highly active during flexion.

In the present study, wrist extensors produced significantly greater muscle activity than the flexors across all experimental conditions. Where the wrist flexors appear to be task dependent, the wrist extensors have a role in joint stability and position accuracy. Previous research suggests that the wrist extensors act to minimize deviations from a target position^5,12,22^. Holmes *et al*. (2015) investigated the effects of wrist perturbations during static gripping tasks and found that the extensors produced the greatest muscle contributions to wrist joint stiffness. In addition, Forman *et al*., 2019 found that during static gripping and wrist exertion tasks, the wrist extensors played a significant role in joint co-contraction for all conditions, where as flexor activity was greatly dependent on task. This relationship–previously observed during static loading–remains consistent during dynamic tasks. Co-contraction is a strategy commonly used to increase joint stiffness and stability. However, it is important to understand that both intrinsic and reflex stiffness contribute to the overall stiffness at any joint^30^. As done in the current study, changes in reflex stiffness can be observed through changes in EMG^30^. However, changes in intrinsic stiffness have not been quantified in this study and findings are therefore referring to alteration in reflexes as a contributor to stiffness.

Anatomical and physiological factors must be considered when examining variations in wrist flexor and extensor activity. The average moment arm (MA) of the wrist flexors is 23% larger than the average MA of the wrist extensors^31^. Additionally, with the exception of FCR and ECR, the physiologic cross-sectional area (PCSA) of the wrist flexors are greater than those of the extensors^21,32^. Together, the MA and PCSA enable the wrist flexors to produce greater moments about the wrist. Finally, these differences result in reduced force production capabilities of the wrist extensors^33^. Therefore, the extensors require greater activation to effectively oppose flexion forces and provide joint stability. Isolated activity of dedicated wrist muscles shows that the wrist flexors’ force vectors have a more direct line of action toward their prime movement than the wrist extensors^34^. FCR produces movement at an angle of 28° above the horizontal; at this angle, 88% of the muscle force is applied toward flexion while 47% of the muscle force is in the radial direction. FCU acts an angle of 40° below the horizontal, resulting in 77% of the muscle force contributing to flexion and 64% acting in the ulnar direction. Conversely, the wrist extensors’ force vectors contribute less toward their prime movement than the wrist flexors. ECR acts at an angle of 70° above the horizontal, resulting in only 34% of muscle force contributing toward extension and 94% in the radial direction. ECU acts at an angle of 71°, providing 33% of muscle force toward extension and 95% toward ulnar deviation. Based on these force vectors, a greater magnitude of extensor activity would be required to balance the flexion force produced by the wrist flexors. The observed disparity in flexor and extensor EMG does not appear to affect task performance when perturbed as wrist kinematics data showed no differences between flexion and extension trials.

### Influence of perturbation direction

In the present study, ECR, EDC, and BR had significantly greater muscle activity during ulnar perturbation trials (Fig. 7). While not significant, FDS, FCU, and ECU produced more muscle activity during radial perturbations than ulnar perturbations, as stated in our third hypothesis. In addition, co-contraction ratios varied based on both phase of movement and perturbation direction (Fig. 8). During flexion, ulnar perturbations produced significantly greater FCR-ECR co-contraction, while radial perturbations produced greater co-contraction during extension. With regard to FDS-EDC co-contraction ratios, radial perturbations produced significantly greater ratios during extension. These ratios indicate that, regardless of movement direction (flexion or extension), ulnar perturbations produced the greatest activity in the wrist extensors. Kinematic tracking data from the WristBot showed that radial perturbations produced significantly greater maximum angular displacement than ulnar perturbations (Fig. 9), despite the same magnitude of perturbation delivered to the hand. However, there were no differences between radial and ulnar perturbations with regard to the time from perturbation onset to maximum angular displacement.

To better understand these results, anatomical and physiological factors must once again be considered. For ulnar perturbations, there was an expected increase in ECR activity, however, there was no similar increase in FCR. As previously stated, the muscle line of action (predominant force vector) for ECR provides a more efficient radial force than that of FCR^34^. Therefore, the CNS may facilitate ECR activity to prioritize a more direct line of action when opposing ulnar perturbations. The increased activity observed from BR is likely due to participants attempting to use elbow flexion force to aid in the resistance of ulnar perturbations. Inverse dynamics suggest that radial/ulnar changes at the wrist joint will influence elbow moments and thus, BR was likely a main contributor to balancing these joint reaction moments^35^. La Delfa and Potvin (2017) found that throughout the majority of an individual’s flexion/extension range of motion, there is a greater wrist moment toward radial deviation than ulnar deviation. Additionally, throughout radial/ulnar deviation, MA has been found to remain relatively linear^36^ and will therefore likely have minimal influence on the results found in the current study. This may account for the greater angular displacement caused by radial perturbations compared to ulnar perturbations. The lack of differences for time to maximum displacement indicates that angular displacement during radial perturbations occurred more rapidly than during ulnar perturbations, suggesting that the wrist ulnar deviators did not increase activity to sufficiently oppose radial perturbations. As previously stated, the wrist extensors function to stabilize the wrist by balancing forces produced by the flexors. Interestingly, we see that FDS produces greater activity during radial perturbations while EDC activity was greater during ulnar perturbations (Fig. 7). Greater FDS activity may be a result of the greater angular displacement produced by radial perturbations, requiring a greater amount of grip force to return to the target trace. However, this may also suggest that during radial trials, EDC does not produce adequate force to balance that of FDS, leading to a reduction in joint stability and increased angular displacement.

### Influence of knowledge

Previous work involving static loading shows that perturbations to the wrist with unknown timing produced significantly greater co-contraction at baseline (Pre-200)^10^. In the present study, all muscles produced less activity during the Pre-200 time period for unknown vs. known trials (Fig. 6). These findings indicate that, during unknown trials, the forearm muscles produced less activity in anticipation of the perturbation. It should be noted that in Holmes *et al*., 2015, participants gripped a hand dynamometer and perturbations caused either wrist flexion or extension. Perturbations occurred during a random time period and no additional tasks were required. In the present study, even during unknown perturbations, participants were still required to track the target cursor. This could suggest a neuromuscular strategy to reduce co-contraction and joint stiffness to improve task performance and instead utilize reflexive muscle activation to oppose perturbations. However, for most muscles, activity during the Pre-15 time period was not different to that of the post-perturbation SLR and LLR time periods. Additionally, no differences were found between known and unknown perturbations for any other time periods. Kinematic data revealed that perturbations with unknown timing produced greater maximum angular displacement (Fig. 8). While not significant, a clear trend in muscle activity was visible for known perturbation timing (Fig. 6). For all muscles, there was an increase in activity from the Pre-200 to Pre-15 time period, followed by a decrease in activity from the Pre-15 to SLR and SLR to LLR time periods. This pattern does not exist for perturbations with unknown timing. For unknown timing an increase is seen from the Pre-200 to Pre-15 time period. However, rather than a subsequent decrease, muscle activity is sustained across the SLR and LLR time periods. This pattern may have emerged as the Pre-15, SLR, and LLR sustain activity to compensate for the lack of anticipatory activity. This pattern is consistent with previous work at the trunk which found that lower pre load activity resulted in greater muscle responses^37,38^. Contrary to our first hypothesis, no differences in anticipatory co-contraction were found between known and unknown perturbations.

Differences seen between the present study and previous perturbation work are likely due, in part, to the nature of the tasks used. In previous work, participants’ wrists were perturbed by a pneumatic device and known perturbations were self initiated by the participant^10^. In the present study, position and timing of known perturbations were provided to the participant, however, the precise timing of the perturbations may have been difficult to anticipate as the location of the perturbation was not marked on the monitor. This difficulty in predicting perturbation onset likely led to increased anticipatory muscle contractions before participants received a perturbation. However, prior to known perturbations, an increase in anticipatory or pre-emptive muscle response was observed and is consistent with previous work at the elbow joint^39^. With regard to unknown timing, however, previous work found a subsequent increase in muscle activity during the reflex period^39,40^. We found no such difference in muscle activity during the SLR or LLR time periods between known and unknown perturbations. These findings suggest that the greater activation during Pre-15, SLR, and LLR compared to Pre-200 time period may not be adequate to oppose perturbing forces. Greater sustained muscle activity following the Pre-15 time period may be required to oppose the greater amount of angular displacement caused by unknown perturbation timing.

### Time period changes

As previously discussed, perturbations with known timing exhibited a consistent trend in muscle activity changes across the four time periods (Fig. 6). Activity during the Pre-200 time period demonstrated the lowest activity followed by an increase during the Pre-15 time period. All muscle activity decreased from Pre-15 to SLR and from SLR to LLR time periods. Perturbations with unknown timing, however, displayed relatively sustained activity following the Pre-200 time period. Holmes *et al*. (2015) saw an increase in co-contraction from baseline to anticipatory period as well as an increase from anticipatory to reflex period in three of five muscle pairings. In the present study, no changes in co-contraction were seen across time periods. In addition, individual muscle activity showed an increase from the Pre-200 time period, however, few differences were found between the Pre-15, SLR, and LLR timer periods. Again, these inconsistencies with previous work are likely due to the differences in experimental set up and that our participants had to remain following a moving target after delivery of the perturbation.

## Conclusions

This is the first study to evaluate radial and ulnar wrist perturbations in combination with a dynamic tracking task. The wrist flexors demonstrated greater task dependency than the wrist extensors. Co-contraction ratios demonstrated significantly greater extensor activity across all experiential conditions. The mechanisms behind these findings are likely due to MA, PCSA, and force vector differences between wrist flexors and extensors. Radial perturbations produced greater angular displacement than ulnar, which is likely due to the relative weakness of ulnar versus radial wrist deviators. Muscle activity during the Pre-200 time period was greater for known than unknown perturbation timing, resulting in greater angular displacement. This work supports a common control strategy seen in previous work^6,22,41^, which demonstrates that an increase in co-contraction, while metabolically inefficient, leads to an inherent increase in joint stiffness necessary to maintain joint stability when encountering perturbing forces. The findings from this study demonstrate that the task dependent nature of the wrist flexors and stabilizing role of the wrist extensors seen during static loading and gripping tasks remains consistent during dynamic movement. To complete any activity of daily living or workplace task, people will require proficient use of their hands and wrists to interact with various objects or tools. This work therefore provides a better understanding of how encountering sudden perturbations may affect performance and injury risk. Receiving an expected (known) perturbation results in increased task performance compared to unexpected (unknown) perturbations, however, this is due to greater anticipatory muscle contractions, which may lead to increased MSD risk. In addition, as the wrist extensors produce relatively higher levels of muscle activity (to stabilize the wrist) regardless of whether or not they are a prime mover, these muscles are susceptible to early-onset of fatigue. The forearm muscular is functionally complex and this study improves our understanding of the roles each muscle group plays in fine motor control of the wrist, providing further support to the stabilization role of the wrist extensors as well as the task dependent nature of the wrist flexors. In addition, the novel aspect of this work includes findings for muscular responses to perturbations during dynamic tasks. This work may be used to inform future applied studies that can have a significant impact on workplace safety.

## References

[1] WSIB. Prevent Musculoskeletal Disorders. (2018).

[2] Van Eerd D (2016). Effectiveness of workplace interventions in the prevention of upper extremity musculoskeletal disorders and symptoms: an update of the evidence. Occup. Environ. Med..

[3] Aarås A, Westgaard RH (1987). Further studies of postural load and musculo-skeletal injuries of workers at an electro-mechanical assembly plant. Appl. Ergon..

[4] Jonsson, B. Kinesiology: with special reference to electromyographic kinesiology. *Electroencephalogr. Clin. Neurophysiol. Suppl*. 417–428 (1978). 285846

[5] De Serres SJ, Milner TE (1991). Wrist muscle activation patterns and stiffness associated with stable and unstable mechanical loads. Exp. Brain Res..

[6] Hogan N (1984). Adaptive control of mechanical impedance by coactivation of antagonist muscles. Autom. Control IEEE Trans. On.

[7] Jacks A, Prochazka A, Trend PS (1988). Instability in human forearm movements studied with feed-back-controlled electrical stimulation of muscles. J. Physiol..

[8] Burdet E, Osu R, Franklin DW, Milner TE, Kawato M (2001). The central nervous system stabilizes unstable dynamics by learning optimal impedance. Nature.

[9] Crevecoeur F, Scott SH, Cluff T (2019). Robust Control in Human Reaching Movements: A Model-Free Strategy to Compensate for Unpredictable Disturbances. J. Neurosci..

[10] Holmes MWR, Tat J, Keir PJ (2015). Neuromechanical control of the forearm muscles during gripping with sudden flexion and extension wrist perturbations. Comput. Methods Biomech. Biomed. Engin..

[11] Forman DA, Forman GN, Robathan J, Holmes MWR (2019). The influence of simultaneous handgrip and wrist force on forearm muscle activity. J. Electromyogr. Kinesiol..

[12] Mogk J, Keir P (2003). The effects of posture on forearm muscle loading during gripping. Ergonomics.

[13] Hägg GM, Öster J, Byström S (1997). Forearm muscular load and wrist angle among automobile assembly line workers in relation to symptoms. Appl. Ergon..

[14] Goodin DS, Aminoff MJ (1992). The basis and functional role of the late EMG activity in human forearm muscles following wrist displacement. Brain Res..

[15] Sinkjær T, Hayashi R (1989). Regulation of wrist stiffness by the stretch reflex. J. Biomech..

[16] Darainy M, Towhidkhah F, Ostry DJ (2007). Control of Hand Impedance Under Static Conditions and During Reaching Movement. J. Neurophysiol..

[17] Gomi H, Osu R (1998). Task-dependent viscoelasticity of human multijoint arm and its spatial characteristics for interaction with environments. J. Neurosci. Off. J. Soc. Neurosci..

[18] Darainy M (2004). Learning to Control Arm Stiffness Under Static Conditions. J. Neurophysiol..

[19] Perreault EJ, Kirsch RF, Crago PE (2002). Voluntary control of static endpoint stiffness during force regulation tasks. J. Neurophysiol..

[20] Franklin DW, Burdet E, Osu R, Kawato M, Milner TE (2003). Functional significance of stiffness in adaptation of multijoint arm movements to stable and unstable dynamics. Exp. Brain Res..

[21] La Delfa, N. J. & Potvin, J. R. A musculoskeletal model to estimate the relative changes in wrist strength due to interacting wrist and forearm postures. *Comput. Methods Biomech. Biomed. Engin*. 1–9, 10.1080/10255842.2017.1366994 (2017). 10.1080/10255842.2017.1366994 28836461

[22] Gribble PL (2003). Role of Cocontraction in Arm Movement Accuracy. J. Neurophysiol..

[23] Wong J, Wilson ET, Malfait N, Gribble PL (2009). Limb Stiffness Is Modulated With Spatial Accuracy Requirements During Movement in the Absence of Destabilizing Forces. J. Neurophysiol..

[24] Masia L, Casadio M, Giannoni P, Sandini G, Morasso P (2009). Performance adaptive training control strategy for recovering wrist movements in stroke patients: a preliminary, feasibility study. Journal of Neuroengineering and Rehabilitation.

[25] Mugnosso M, Marini F, Holmes M, Morasso P, Zenzeri J (2018). Muscle fatigue assessment during robot-mediated movements. J. NeuroEngineering Rehabil..

[26] Perotto, A. O. *Anatomical Guide for the Electromyographer: The Limbs and Trunk*. (1994).

[27] Forgaard CJ, Franks IM, Maslovat D, Chua R (2016). Perturbation Predictability Can Influence the Long-Latency Stretch Response. PloS One.

[28] Holmes MWR, Keir PJ (2014). Muscle Contributions to Elbow Joint Rotational Stiffness in Preparation for Sudden External Arm Perturbations. J. Appl. Biomech..

[29] Pruszynski JA, Scott SH (2012). Optimal feedback control and the long-latency stretch response. Exp. Brain Res..

[30] Guarin DL, Kearney RE (2018). Unbiased Estimation of Human Joint Intrinsic Mechanical Properties During Movement. IEEE Trans. Neural Syst. Rehabil. Eng..

[31] Gonzalez RV, Buchanan TS, Delp SL (1997). How muscle architecture and moment arms affect wrist flexion-extension moments. J. Biomech..

[32] Lemay MA, Crago PE (1996). A dynamic model for simulating movements of the elbow, forearm, and wrist. J. Biomech..

[33] Hallbeck MS (1994). Flexion and extension forces generated by wrist-dedicated muscles over the range of motion. Appl. Ergon..

[34] Bawa P, Chalmers GR, Jones KE, Søgaard K, Walsh ML (2000). Control of the wrist joint in humans. Eur. J. Appl. Physiol..

[35] van Ingen Schenau GJ, Boots PJM, de Groot G, Snackers RJ, van Woensel WWLM (1992). The constrained control of force and position in multi-joint movements. Neuroscience.

[36] Horii E, An KN, Linscheid RL (1993). Excursion of prime wrist tendons. J. Hand Surg..

[37] Eriksson Crommert AEM, Thorstensson A (2009). Trunk muscle reactions to sudden unexpected and expected perturbations in the absence of upright postural demand. Exp. Brain Res..

[38] Stokes IAF, Gardner-Morse M, Henry SM, Badger GJ (2000). Decrease in Trunk Muscular Response to Perturbation With Preactivation of Lumbar Spinal Musculature. Spine.

[39] Holmes MWR, Keir PJ (2012). Posture and hand load alter muscular response to sudden elbow perturbations. J. Electromyogr. Kinesiol..

[40] Latash ML (1994). Control of fast elbow movement: a study of electromyographic patterns during movements against unexpectedly decreased inertial load. Exp. Brain Res..

[41] Milner TE (2002). Adaptation to destabilizing dynamics by means of muscle cocontraction. Exp. Brain Res..

